# A cross-sectional study on resilience, anxiety, depression, and psychoactive substance use among heterosexual and sexual minority adolescents in Nigeria

**DOI:** 10.1186/s12889-023-16660-1

**Published:** 2023-09-09

**Authors:** Olanrewaju Ibikunle Ibigbami, Olakunle Ayokunmi Oginni, Cory Bradley, Joanne Lusher, Nadia Adjoa Sam-Agudu, Morenike Oluwatoyin Folayan

**Affiliations:** 1 Department of Mental Health, Obafemi Awolowo University, Ile-Ife, Nigeria; 2 Division of Infectious Diseases, Department of Medicine, Washington University School of Medicine in St. Louis, St. Louis, MO USA; 3 Regent’s University London, London, UK; 4 International Research Center of Excellence, Institute of Human Virology Nigeria, Abuja, Nigeria; 5 Institute of Human Virology, University of Maryland School of Medicine, Baltimore, USA; 6 Department of Child Dental Health, Obafemi Awolowo University, Ile-Ife, Nigeria; 7 Oral Health Initiative, Nigerian Institute of Medical Research, Yaba, Lagos, Nigeria

**Keywords:** Adolescents, Anxiety, Depression, Alcohol, Multiple substance use

## Abstract

**Background:**

Mental health-related problems predispose alcohol and other psychoactive substances use as coping strategies. We assessed associations between resilience and anxiety symptoms, depressive symptoms, problematic alcohol, and multiple psychoactive substance use among sexual minority and heterosexual adolescents in Nigeria.

**Methods:**

This was a secondary analysis of a subset of data generated through an online cross-sectional study conducted between 16^th^ and 31^st^ of October 2020. Data extracted for adolescents in Nigeria age 13–19 years were: dependent variables (alcohol use using the CAGE test, multiple psychoactive substance use, depressive symptoms using the Patient Health Questionnaire-9, and anxiety symptoms using the Generalized Anxiety Disorder-7 measure); independent variables (resilience using the Connor-Davidson resilience scale and sexual identity), and confounding factors (age and sex). Associations between dependent and independent variables were determined using multivariable logistic regression analyses after controlling for confounders.

**Results:**

Of the 1419 adolescent participants, 593 (42%) were sexual minority individuals, 533 (37.6%) had high depressive symptoms, 381 (26.8%) had high anxiety symptoms, 177 (12.5%) had problematic alcohol use and 389 (27.4%) used multiple psychoactive substances. Resilience was significantly associated with lower odds of anxiety (AOR:0.96, 95% CI: 0.94–0.97, *p* < 0.001) and depressive (AOR:0.94, 95% CI: 0.92–0.96, *p* < 0.001) symptoms, problematic alcohol use (AOR:0.97, 95% CI: 0.95–0.99, *p* = 0.002), and multiple psychoactive substance use (AOR:0.95, 95% CI: 0.93–0.96, *p* < 0.001). Sexual minority adolescents had significantly higher odds of anxiety (AOR:4.14, 95% CI: 3.16–5.40, *p* < 0.001) and depressive symptoms (AOR:4.79; 95% CI: 3.73–6.15, *p* < 0.001), problematic alcohol use (AOR:2.48, 95% CI: 1.76–3.49, *p* < 0.001), and multiple psychoactive substance use (AOR:5.69, 95% CI: 4.34–7.47, *p* < 0.001).

**Conclusion:**

Sexual minority adolescents and adolescents with low resilience have a higher need for interventions to reduce the risk of anxiety, depression, and the use of alcohol and other psychoactive substances.

## Introduction

Sexual minority individuals face a higher likelihood of experiencing discrimination, limited access to public health resources, and increased vulnerability to violence [1–3]. Acts of violence against sexual minorities can stem from biased attitudes towards their sexual orientation [4], particularly affecting those living with HIV and individuals forced into marriage [5]. Harmful societal attitudes and norms rooted in patriarchal heterosexism and laws criminalizing same-sex relationships further amplify the risk of harassment and violence faced by sexual minority individuals [5, 6].

Harassment and violence can have detrimental effects on mental health, leading to loneliness and feelings of depression [7, 8]. These mental health challenges significantly impact the overall well-being and quality of life of affected individuals [9, 10]. As a result, some individuals may turn to alcohol and other psychoactive substances as coping mechanisms [11]. For sexual minority adolescents, the experience of exclusion can be particularly difficult, especially as they navigate their own sexual identities. They may have to deal with low self-esteem [12], mood disorders such as major depressive disorder and dysthymic disorder, anxiety disorders such as generalized anxiety disorder, agoraphobia, and panic disorder, as well as substance use disorders involving alcohol, tobacco, marijuana, and illicit drug abuse and dependence [13–15]. Due to parental resistance and limited support for sexual minority populations, these adolescents may face even greater challenges in accessing the services and care they need [16]. In Nigeria, for example, adolescents are required to obtain parental consent for healthcare services [17], and open discussions about sex and sexuality are often considered taboo [18, 19]. Additionally, intense homophobia in Nigeria may further hinder sexual minority adolescents from seeking appropriate help when they encounter mental health issues.

Resilience refers to an individual's capacity to maintain or restore optimal mental health and functioning even in the face of adversities [20]. It plays a crucial role in mitigating the negative effects of social discrimination. Individuals with higher levels of resilience are less likely to experience poor mental health and well-being in adversity, compared to those with lower resilience [21]. Building resilience can be facilitated by a supportive environment that provides necessary resources and social support [22]. However, innate, and environmental factors such as social discrimination can undermine resilience and render individuals more vulnerable to mental health challenges [23].

Prior studies indicate that sexual minority adolescents have higher odds of reporting lower resilience, depressive and anxiety symptoms, problematic alcohol use, and the use of multiple psychoactive substances compared to heterosexual adolescents [11, 24, 25], However, none of these studies were conducted in African countries, where social and cultural contexts differ. Therefore, investigating these associations within the unique cultural landscape of sub-Saharan Africa, specifically Nigeria, is crucial for a comprehensive understanding of the experiences of sexual minority adolescents in this region.

Country-specific studies are essential in mental health, as culture plays a moderating role in mental health outcomes [26], resilience [27], and the adoption of coping strategies [28], although the relationships between these variables are complex. Cultural factors significantly influence vulnerability to psychological and behavioral problems [29, 30], and can shape the adoption of maladaptive coping mechanisms such as alcohol use. Thus, it is important to understand how sexual minority individuals Nigeria may respond differently to the experience of sexual minority stress.

To guide study design, we applied a framework based on the minority stress model. According to this model, minority populations, including sexual minority adolescents [31], face additional stressors and challenges compared to the general population [32]. Previous research has already established links between resilience and depressive and anxiety symptoms, problematic alcohol use, and the use of multiple psychoactive substances [33–36]. In our previous work, we have also observed that sexual minority adolescents in Nigeria tend to experience poorer mental health outcomes compared to their heterosexual counterparts [24].

The main aim of our study was to assess for associations between resilience and anxiety symptoms, depressive symptoms, problematic alcohol use, and multiple psychoactive substance use among both sexual minority and heterosexual adolescents in Nigeria.

Our hypotheses were twofold: first, we hypothesized that sexual minority adolescents would have a higher likelihood of experiencing anxiety and depressive symptoms, engaging in problematic alcohol use, and using multiple psychoactive substances compared to heterosexual adolescents. Second, we hypothesized that adolescents with lower levels of resilience would be more likely to exhibit symptoms of anxiety and depression, engage in problematic alcohol use, and use multiple psychoactive substances.

## Methods

### Ethical considerations

The study received ethical approval from the Health Research Ethics Committee of the Institute of Public Health at the Obafemi Awolowo University in Ile-Ife, Nigeria (Approval No: IPHOAU/12/1571). As part of the approval process, a waiver of parental consent was granted for adolescents aged 13–17 years, considering the sensitive nature of the topic related to sexual identity. This waiver aligned with national guidelines on conducting sexual and reproductive health research involving adolescents [37]. The research adhered to the principles outlined in the Declaration of Helsinki (2013 revision) [38].

Participants provided informed consent by checking a box to indicate their willingness to participate in the study. To ensure confidentiality, all data collected were irreversibly anonymized, meaning that no personally identifiable information was linked to the data. As a token of appreciation for their participation, all participants were offered a compensation of N100 (approximately $0.27) to cover internet usage costs.

### Study design

The present study is a secondary analysis of the dataset of a cross-sectional online study conducted between 16th and 31st October 2020. An online electronic survey platform, specifically SurveyMonkey®, was used to collect study data.

### Study population and study site

For the parent study, adolescents and adults aged 13 years and above were recruited from all 36 states and the Federal Capital Territory in Nigeria. The current analysis focuses on a sub-group of participants aged 13 to 19 years. Details of the parent study and methods have been previously published [24].

### Sample size

For this sub-study, a pre-survey minimum sample size of 50 valid respondents was established for each of state and the Federal Capital Territory. This translates to a minimum sample size requirement of 1,850 participants in total. From a statistical modelling perspective, this sample was adequate, as a minimum of 10 participants per independent variable was required to conduct regression analyses with a significance level (*p*-value) of 0.05 [39].

### Sampling procedures

A non-probability sampling method was employed. A total of 29 peer educators and staff from a non-governmental organization (Total Health Empowerment and Development Initiative (THEDI)) played a crucial role in disseminating the study link to their networks of sexual minority individuals and the public. Initial participants were encouraged to further share the survey link with their contacts. The link was also actively shared on social media platforms such as Facebook, Twitter, and Instagram, as well as through network email lists and WhatsApp groups. Collaborating non-governmental organizations operating across Nigeria were also requested to distribute the survey link within their networks to support the recruitment process.

To accommodate individuals with low literacy, an open link was made available at THEDI offices, as well as its partner organizations' offices. This allowed individuals to complete the survey with assistance from peer educators. The questionnaire was conducted in English and required an average of 15 min to complete. Each participant was only allowed to submit a single questionnaire on their device and had the option to edit their answers before final submission. To prevent multiple entries, participants were restricted to one entry per IP address.

### Independent study variables

Resilience was measured using the 10-item Connor-Davidson Resilience Scale [40]. Participants rated each item on a five-point Likert scale, ranging from 0 ("Not true at all") to 4 ("True nearly all the time"). The total scores ranged from 0 to 40, with higher scores indicating greater resilience. The scale had been previously administered to a sample of Nigerians [41], and in this study, the Cronbach's alpha coefficient for the scale was 0.87.

Information regarding sexual identity was collected, allowing participants to indicate their sexual orientation as heterosexual, gay, lesbian, bisexual, or “prefer not to say”. For data analysis, individuals who self-identified as gay, lesbian, or bisexual were classified as sexual minority individuals by selecting the corresponding checkbox. Those who chose not to disclose their sexual identity (N = 38) were excluded from the data analysis.

### Dependent study variables

#### Alcohol abuse

Problematic alcohol use was assessed using the Cut, Annoyed, Guilty, and Eye-opener (CAGE) test [42, 43]. The CAGE consists of four yes-or-no questions, and individuals with a score of 2 or more were identified as having drinking problems. The CAGE test has demonstrated high test–retest reliability (0.80–0.95) and satisfactory correlations (0.48–0.70) with other measures of alcoholism [44]. In this sample, the Cronbach's alpha coefficient for the CAGE test was 0.86.

#### Multiple psychoactive substance use

For the assessment of multiple psychoactive substance use, participants were asked about their use of psychoactive substances other than alcohol. Response options ranged from "never used" (0) to "regularly used" (3). Each psychoactive substance was coded as either “not used” (0) or “used” (1). Participants who reported using two or more psychoactive substances were classified as multiple psychoactive substance users.

#### Depressive symptoms

Depressive symptoms were measured using the Patient Health Questionnaire (PHQ-9) [45]. The PHQ-9 consists of nine questions assessing the frequency of depressive symptoms, with response options ranging from "not at all" (0) to "nearly every day" (3). Scores on the PHQ-9 can range from 0 to 27, with different score ranges indicating varying levels of depressive symptoms. In this study, the PHQ-9 scores were dichotomized into the absence/mild depressive symptoms category and the moderate/severe depressive symptoms category. The PHQ-9 has shown a test–retest reliability score of 0.89 among young Nigerian adults [46]. The Cronbach's alpha coefficient for the PHQ-9 in this sample was 0.93.

#### Generalized anxiety symptoms

Anxiety symptoms were assessed using the seven-item Generalized Anxiety Disorder-7 (GAD-7) measure [47]. Participants rated the frequency of experiencing seven core anxiety symptoms over the past two weeks, with response options ranging from "not at all" (0) to "nearly every day" (3). The total score on the GAD-7 can range from 0 to 21. For the regression analyses, scores were categorized as low (0–9) or high (10 and above) anxiety. The GAD-7 demonstrated good internal consistency in this sample, with a Cronbach's alpha coefficient of 0.91.

### Confounders

The potential confounding variables considered in this study were age at last birthday (in years) and sex assigned at birth (male, female). Age was included as a confounding variable due to its established associations with alcohol abuse and psychoactive substance use [48, 49], depressive symptoms [50], generalized anxiety symptoms [51], resilience [52], and sexual identity [53]. Similarly, sex assigned at birth was considered as a confounding variable because it has been found to be significantly associated with alcohol abuse and psychoactive substance use [54], depressive symptoms [55], generalized anxiety symptoms [56, 57], resilience [58], and sexual identity [59].

### Data analysis

Descriptive statistics were computed for all variables, including means and standard deviations for continuous variables and frequencies and percentages for categorical variables. Chi-square tests or t-tests were used to examine the associations between the dependent, independent, and confounding variables. Four separate multivariable logistic regression models were constructed to analyze the associations between the independent variable and each of the four dependent variables while controlling for confounding variables. Regression coefficients, along with their corresponding 95% confidence intervals (CI) and p-values, were calculated using IBM SPSS Statistical Software (Version 23).

## Results

A total of 1,419 adolescents' data were analyzed in this study, with a mean age of 17.10 (± 1.61) years, and a majority (64.0%) being assigned male at birth. Among the participants, 363 (25.6%) identified as gay, 130 (9.2%) as lesbian, and 100 (7%) as bisexual. The mean resilience score was 19.19 (± 7.94). In terms of mental health outcomes, 533 (37.6%) participants reported high depressive symptoms, 381 (26.8%) reported high anxiety symptoms, 177 (12.5%) had problematic alcohol use, and 389 (27.4%) reported using multiple psychoactive substances.

Table 1 shows that a significantly larger percentage of sexual minority compared to heterosexual adolescents (58.2% vs 22.8%; *p* < 0.001), experienced symptoms of depression. Furthermore, adolescents with depressive symptoms reported lower levels of resilience (20.9 vs 16.4; *p* < 0.001). Similarly, a significantly higher proportion of sexual minority in comparison to heterosexual adolescents, reported symptoms of anxiety (42.8% vs 15.4%; *p* < 0.001); and adolescents with anxiety symptoms demonstrated lower levels of resilience (20.2 vs 17.1; *p* < 0.001). Moreover, a significantly greater percentage of sexual minority adolescents displayed problematic alcohol use, compared to their heterosexual counterparts (19.4% vs 7.5%; *p* < 0.001), and adolescents with problematic alcohol use reported lower levels of resilience (19.5 vs 16.9; *p* < 0.001). Additionally, a significantly larger proportion of sexual minority adolescents reported using multiple psychoactive substances (48.7% vs 12.1%; *p* < 0.001), and adolescents using multiple psychoactive substances reported lower levels of resilience (20.3 vs 16.3; *p* < 0.001).

**Table 1:** Differences in the proportion of adolescents 13 to 19 years old in Nigeria who experience anxiety, depressive symptoms, anxiety symptoms, problems with alcohol use and multiple psychoactive substance use (*N* = 1419)

Variables	Total n (%)	Depressive symptoms			Anxiety symptoms			Problem with alcohol use			Multiple psychoactive substance use		
		**No** ***N*** = **886** **n (%)**	**Yes** ***N*** = **533** **n (%)**	**t/χ**^**2**^ **(*p* value)**	**No** ***N*** = **1038** **n (%)**	**Yes** ***N*** = **381** **n (%)**	**t/χ**^**2**^ **(*p* value)**	**No** ***N*** = **1242** **n (%)**	**Yes** ***N*** = **177** **n (%)**	**t/ χ**^**2**^ **(*p* value)**	**Less than 2 or none** ***N* = 1030** **n (%)**	**2 or more** ***N* = 389** **n (%)**	**t/ χ**^**2**^ **(*p* value)**
**Age in years** mean (SD)	17.1 (1.6)	17.23(1.4)	16.9 (1.9)	87.2 (< 0.001)	17.2 (1.5)	16.9 (1.8)	41.2 (0.001)	17.1 (1.62)	17.4 (1.53)	0.3 (0.008)	17.0 (1.6)	17.4 (1.5)	0.004 (< 0.001)
**Sex at birth**													
Male	908 (64.0)	556 (61.2)	352 (38.8)	1.56 (0.231)	649 (71.5)	259 (28.5)	3.60 (0.058)	782 (86.1)	126 (13.9)	4.6 (0.03)	645 (71.0)	263 (29.0)	3.05 (0.083)
Female	511 (36.0)	330 (64.6)	181 (35.4)		389 (76.1)	122 (23.9)		460 (90.0)	51 (10.0)		385 (75.3)	126 (24.7)	
**Sexual orientation**													
Heterosexual	826 (58.2)	638 (77.2)	188 (22.8)	84.64 (< 0.001)	699 (84.6)	127 (15.4)	132.5 (< 0.001)	764 (92.5)	62 (7.5)	44.7 (< 0.001)	726 (87.9)	100 (12.1)	232.7 (< 0.001)
Sexual minority^a^	593 (41.8)	248 (41.8)	345 (58.2)		339 (57.2)	254 (42.8)		478 (80.6)	115 (19.4)		304 (51.3)	289 (48.7)	
**Resilience** mean (SD)	19.2 (7.9)	20.9 (8.7)	16.4 (5.4)	111.28 (< 0.001)	20.2 (8.4)	17.1 (6.8)	55.7 (< 0.001)	19.5 (7.9)	16.9 (8.1)	0.2 (< 0.001)	20.3 (8.2)	16.3 (6.5)	25.5 (< 0.001)

^a^Gay, lesbian, or bisexual

Table 2 presents multivariate logistic regression of associations between resilience and depressive and anxiety symptoms, problematic alcohol and multiple psychoactive substance use, indicating the odds ratios (AOR) and confidence intervals (CI) for various factors. Compared to heterosexual adolescents, sexual minority adolescents had significantly higher odds of experiencing depressive symptoms (AOR: 4.79; 95% CI: 3.73–6.15, *p* < 0.001), as did individuals with lower resilience (AOR: 0.94; 95% CI: 0.92–0.96, *p* < 0.001). Similarly, sexual minority individuals had significantly higher odds of experiencing anxiety symptoms (AOR: 4.14; 95% CI: 3.16–5.40, *p* < 0.001), as did individuals with lower resilience (AOR: 0.96; 95% CI: 0.94–0.97, *p* < 0.001).

**Table 2:** Multivariate logistic regression analysis of associations between resilience and depressive symptoms, anxiety symptoms, problem with alcohol use, multiple psychoactive substance use, and resilience among adolescents 13 to 19 years old in Nigeria (*N* = 1419)

Variables	Depression AOR (95% CI)	*p*-value	Anxiety AOR (95% CI)	*p*-value	Problem with alcohol use AOR (95% CI)	*p*-value	Multiple psychoactive substance use	*p*-value
**Age in years**	0.81 (0.75–0.87)	< 0.001	0.83 (0.76–0.89)	< 0.001	1.12 (1.00–1.24)	0.047	1.15 (1.05–1.25)	0.002
**Sex at birth**								
Female (ref)	1.00	-	1.00	-	1.00	-	1.00	-
Male	1.08 (0.84- 1.39)	0.538	1.19 (0.91–1.56)	0.195	1.37 (0.97–1.95)	0.078	1.13 (0.86—1.48)	0.397
**Sexual orientation**								
Heterosexual (ref)	1.00	-	1.00	-	1.00	-	1.00	-
Sexual minority	4.79 (3.73–6.15)	< 0.001	4.14 (3.16–5.40)	< 0.001	2.48 (1.76–3.49)	< 0.001	5.69 (4.34–7.47)	< 0.001
**Resilience**	0.94 (0.92–0.96)	< 0.001	0.96 (0.94- 0.97)	< 0.001	0.97 (0.95–0.99)	0.002	0.95 (0.93–0.96)	< 0.001
Omnibus test of coefficients	265.80	< 0.001	187.92	0.021	59.32	< 0.001	280.70	< 0.001
Nagelkerke R^2^	0.248	**-**	0.180	**-**	0.077	**-**	0.260	**-**

Regarding problematic alcohol use, sexual minority individuals had significantly higher odds (AOR: 2.48; 95% CI: 1.76–3.49, *p* < 0.001), as did individuals with lower resilience (AOR: 0.97; 95% CI: 0.95–0.99, *p* = 0.002). Moreover, sexual minority individuals had significantly higher odds of using multiple psychoactive substances (AOR: 5.69; 95% CI: 4.34–7.47, *p* < 0.001), as did individuals with lower resilience (AOR: 0.95; 95% CI: 0.93–0.96, *p* < 0.001).

## Discussion

Our findings revealed high rates of mental health challenges and substance use among adolescents in Nigeria. One in three adolescents experienced high depressive symptoms, one in four had high anxiety symptoms, one in four engaged in multiple psychoactive substance use, and one in six exhibited problematic alcohol use. Sexual minority individuals were found to be at a significantly higher risk for depression, anxiety, problematic alcohol use, and multiple psychoactive substance use compared to heterosexual adolescents. Additionally, lower levels of resilience were associated with increased odds of experiencing mental health challenges and engaging in maladaptive coping strategies. These findings support our study hypotheses.

This is the first study assessing associations between resilience and depressive and anxiety symptoms, and psychoactive substance use among adolescents in Nigeria. A notable strength of our study was the nationally representative sample of adolescents, allowing for the generation of context-specific information. The inclusion of a large sample of sexual minority adolescents facilitated robust sub-analysis and the generation of results with policy and practice implications for this specific population.

However, there are a few limitations. Data collection was conducted online and in English, which excluded individuals with low literacy levels or limited access to smartphones and the internet. Moreover, the use of non-probability sampling techniques restricts the generalizability of the findings. Furthermore, the cross-sectional study design limits causal inferences, and the analysis did not include gender identity, focusing instead on sex assigned at birth. In addition, the study used screening instruments to measure the mental health problems and problematic psychoactive substance use; this may have led to the higher prevalence of the disorders. Despite these limitations, the study provides valuable insights to inform the development of programs aimed at reducing mental health risks and maladaptive coping strategies among adolescents, including sexual minority individuals.

Chronic exposure to stress, including that faced by sexual minority individuals, is physiologically and or emotionally challenging, resulting in the activation of stress responses and adaptive processes to regain homeostasis [60–62]. Often, people experiencing emotional and physiological stress turn to alcohol and other psychoactive substances as coping strategies [63]. The regular and binge use of psychoactive drugs can act as pharmacological stressors [64]. When individuals experience stress, their bodies activate stress responses and initiate adaptive processes to restore balance and stability [63]. The level of adaptability plays a crucial role, as lower adaptability is associated with stronger stress responses and an increased risk of persistent dysregulation of homeostasis [61]. Therefore, factors such as the intensity, controllability, predictability, mastery, and adaptability are crucial in understanding how stress contributes to the heightened risk of engaging in maladaptive behaviors such as problematic use of alcohol and multiple psychoactive substance use [65, 66]. One of the stress responses is the triggering of the release of cortisol and adrenocorticotropic hormone [67]. Cortisol interacts with the brain's reward system, contributing to the reinforcing effects of alcohol and other psychoactive substances, and increasing the motivation to consume more alcohol and other psychoactive substances to achieve similar effects [67]. Moreover, cortisol promotes habit-based learning by impacting learning and memory processes, which can ultimately lead to the development of problematic drinking and psychoactive substance use behaviors [68].

This study’s findings are important, as Nigeria is a particularly stressful country [69] with a high proportion of adolescents experiencing adverse childhood events [70]. Chronic exposure to stress may increase the risk for addiction. High levels of stress and associated high cortisol levels are also linked to the development of mental health disorders such as depression [71]. This may explain the high rate of depression in our study sample. This risk of a vicious cycle of stress, addiction and depression is especially more likely for sexual minority individuals who live in an intensely homophobic environment like Nigeria [72]. Thus, a high proportion of adolescents in Nigeria, especially sexual minority adolescents, is at risk of substance addiction problems if interventions are not put in place now to address their stress and mental health issues.

Although our study did not specifically examine the association between sexual orientation and resilience, previous research has indicated that sexual minority individuals face barriers in accessing assets and resources that contribute to resilience [73]. Moreover, the misuse of psychoactive substances further exacerbates the psychological distress resulting from experiences of social discrimination [74].

We initially anticipated that the experiences of sexual minority individuals in Nigeria would differ from those in other cultures, considering the influence of culture on resilience [75–77]. Surprisingly, it appears that cultural nuances have less impact on how adolescents respond to drivers of anxiety and depression, as our findings align with the experiences of adolescents in Europe and America [26, 27, 78]. Unfortunately, we were unable to locate another study conducted with an indigenous African population, which limits our ability to compare intra-cultural differences or similarities with our findings. Future research endeavors should delve into exploring how culture influences the impact of mental health experiences among adolescents.

Our study, like other studies, also suggests that heterosexual adolescents turn to alcohol and multiple psychoactive substances to cope with depression and anxiety [79, 80]. However, it is important to recognize that the challenges faced by heterosexual adolescents leading to the adoption of maladaptive coping strategies may differ from those experienced by sexual minority adolescents. Therefore, further research is needed to identify the specific causes of chronic stress within diverse populations of adolescents in Nigeria. These findings have the potential to inform the development of targeted interventions that are culturally tailored, and aimed at reducing the use of maladaptive coping mechanisms. Enhancing resilience is a promising approach in mitigating this risk, as resilience can be taught and acquired [81, 82]. By incorporating resilience-building strategies into intervention programs for adolescents, particularly for sexual minority individuals in Nigeria, we may effectively promote better mental health outcomes.

In conclusion, the study findings highlight that sexual minority adolescents were more susceptible to anxiety and depressive symptoms, problematic alcohol use, and multiple psychoactive substance use compared to heterosexual adolescents. Additionally, adolescents with lower levels of resilience were more prone to experiencing symptoms of anxiety and depression, engaging in problematic alcohol use, and using multiple psychoactive substances. Based on these results, we recommend that organizations working with adolescents in Nigeria, particularly those who identify as sexual minorities, incorporate resilience-building strategies into their strategic engagement plans to address the mental health challenges and risk behaviors observed in this population.

## Data Availability

The datasets used and/or analysed during the current study are available from the corresponding author upon reasonable request.

## Abbreviations

AOR: Adjusted Odds Ratio
CI: Confidence Interval
SD: Standard Deviation
